# Research advances in reproduction for dairy goats

**DOI:** 10.5713/ajas.19.0486

**Published:** 2019-07-18

**Authors:** Jun Luo, Wei Wang, Shuang Sun

**Affiliations:** 1 Shaanxi Key Laboratory of Molecular Biology for Agriculture, College of Animal Science and Technology, Northwest A&F University, Yangling, Shaanxi 712100, China; 2 College of Animal Science and Veterinary Medicine, Shenyang Agricultural University, Shenyang, Liaoning 110866, China

**Keywords:** Dairy Goat, Reproduction, Reproductive Physiology, Estrus Synchronization (ES), Artificial Insemination (AI), Embryo Transfer (ET)

## Abstract

Considerable progress in reproduction of dairy goats has been made, with advances in reproductive technology accelerating dairy goat production since the 1980s. Reproduction in goats is described as seasonal. The onset and length of the breeding season is dependent on various factors such as breed, climate, physiological stage, male effect, breeding system, and photoperiod. The reproductive physiology of goats was investigated extensively, including hypothalamic and pituitary control of the ovary related to estrus behavior and cyclicity etc. Photoperiodic treatments coupled with the male effect allow hormone-free synchronization of ovulation, but the kidding rate is still less than for hormonal treatments. Different protocols have been developed to meet the needs and expectations of producers; dairy industries are subject to growing demands for year round production. Hormonal treatments for synchronization of estrus and ovulation in combination with artificial insemination (AI) or natural mating facilitate out-of-season breeding and the grouping of the kidding period. The AI with fresh or frozen semen has been increasingly adopted in the intensive production system, this is perhaps the most powerful tool that reproductive physiologists and geneticists have provided the dairy goat industry with for improving reproductive efficiency, genetic progress and genetic materials transportation. One of the most exciting developments in the reproduction of dairy animals is embryo transfer (ET), the so-called second generation reproductive biotechnology following AI. Multiple ovulation and ET (MOET) program in dairy goats combining with estrus synchronization (ES) and AI significantly increase annual genetic improvement by decreasing the generation interval. Based on the advances in reproduction technologies that have been utilized through experiments and investigation, this review will focus on the application of these technologies and how they can be used to promote the dairy goat research and industry development in the future.

## INTRODUCTION

In the era of scarcity of goods and materials, the dairy goat was called the “poor man’s cow”. The traditional breeding method of dairy goats was used to provide impoverished farmers and herdsmen with limited income sources. Dairy goats can provide high-quality animal products such as milk, meat, leather, manure and so on, and create a large avenue for farmers and industries, goat milk with small fat globules and diversified nutrients is unanimously considered by the nutritionists as the dairy products similar to human milk and is also known as the “king of milks” [1]. The nutritional health and therapeutic effects of goat milk have been recorded in ancient Chinese classic medicine literature, and the current Chinese dairy goat industry is in the golden period of making a significant progress and incomes [2]. Dairy goats have increasingly become a profitable business for farmers in developing countries and the nutritious functional food for consumers in the world, which has created a great opportunity for dairy goat industry and producers, especially in Asia and Africa.

The knowledge of ruminant reproduction dates back to 300BC, the period from 1666 to 1676 has been designated a decade of discovery based on the following observations: i) it was proposed that ovaries contain eggs, ii) Regnier De Graff hypothesized that ovarian follicles contain eggs, iii) the corpus luteum was named, De Graff reported that corpora were transient, provided an estimation of the number of embryos in the uterus, and disappeared following parturition, and iv) spermatozoa were first visualized by Hamm and Leeuenhoek [3,4]. The modern dairy goat has been selected and managed for high milk production and efficiency. It is particularly challenging to overcome the intrinsic seasonality of estrus in dairy goats, but the major advancements have been made that are operational, increasing understanding of the underlying processes of reproductive physiology, and seasonal breeding of dairy goat has resulted in the gradual development of reproductive technologies.

Artificial insemination (AI) is probably the most important biotechnology for significant genetic improvement used in the dairy goats. It entails semen collection, processing, and evaluation, with an emphasis on bucks. Nowadays, AI is extensively used in intensive dairy goat production systems. In the last decades, the sex-sorted sperm technique has opened a new window to improve the reproductive efficiency of dairy goat to produce kids of the same sex, although some technique aspects require further research attention.

Embryo transfer (ET) experiments have demonstrated the critical importance of the state of the uterine environment to embryo viability and that the embryo must be present in the uterus in post-estrus does to prevent corpus luteum regression. The ET between breeds of differing gestational length has indicated conclusively that the genotype determines the duration of pregnancy. Because there is anenormous amount of information concerning the reproduction of dairy goats, focus will be on the reproductive physiology of males and females, semen cryopreservation, sex-sorted sperm, estrus synchronization (ES), AI, embryo biotechnologies, ET, and other recent discoveries.

## REPRODUCTIVE PHYSIOLOGY

The reproductive activity of dairy goats is controlled by various factors both *in vivo* and *in vitro*. Among them, the hypothalamic-pituitary-gonadal axis plays a major role in regulation, with the hypothalamus modulating the secretion of pituitary gonadotropins or inhibitory factors. Gonadotropin acts on the gonad through the peripheral blood circulation, Gonadotropin can control the individual’s development and reproductive process through affecting the exocrine and endocrine glands [5]. Most dairy goats are single-fetal mammals, with 1 to 3 mature follicles per estrus cycle, while other follicles of dairy goats are inhibited into atresia and apoptosis. According to classical reproductive physiology theory, the formation and development of follicles and the occurrence of ovulation depend entirely on the combined effects of follicle stimulating hormone (FSH) and luteinizing hormone (LH). The selection of dominant follicles basically depends on two aspects: one is the level of gonadotropin in blood, and the other is the expression of hormone receptors in follicles [6]. FSH is an important regulatory factor in the development of follicles, stimulating development of follicles and the recruitment of immature eggs, and plays an important role in the regulation of the balance of follicular recruitment and atresia. The LH controls the development, selection, and ovulation of the dominant follicle, and also induces apoptosis of other small follicles. Under physiological conditions, when FSH rises up to a threshold, follicles of 2 to 5 mm in diameter could be recruited periodically, the duration time of follicle recruitment determines the number of follicle recruited [7].

During the dominant follicle selection process of singleton animals, because of the different sensitivity of follicles to the elevation of gonadotropin concentration, several sensitive follicles are selected from the follicle pool. Through the effect of FSH, these sensitive follicles increase the number of granulose cells and follicular fluid, which results in the growth of follicles. The recruitment, development, selection, and ovulation of follicles are the primary factors affecting the fertility of goats [8]. In the process of follicular development, the recruitment of primordial follicles, development of preantral follicles, selection of antral follicles, and maturation and ovulation of dominant follicles are key factors that determine the number and quality of eggs; these factors play key roles in determining the pregnancy rate and litter size in goats. Previously it was found that several signaling pathways participate in the process of follicular development. These pathways include Wnt/β-catenin, Nodal mammalian target of rapamycin (mTOR), and bone morphogenetic protein (BMP)/Smad [9]. A study in Israel indicated that a high level of milk production has a significant adverse effect on reproductive performance [10]. The heritability of litter size was very low (~0.05), and the genetic correlation with milk production was also low (0.0 to 0.2) [11–13]. Delivery in non-breeding season may be practical for all year around production system; therefore, the promotion of advance breeding techniques is necessary in the dairy goat breeding industry [14].

As a seasonal breeder, dairy goat females naturally have a period of anoestrus during the spring in the northern hemisphere, and males also display wide changes in sexual activity. The breeding season of dairy bucks begins in the early autumn and ends in late winter. The sexual behavior, testicular size, an index of the spermatogenetic activity, and the quantitative and qualitative sperm production of bucks decrease dramatically during the non-breeding season [8]. Appraisal of the reproductive functions of dairy goat bucks is usually performed between 7 and 9 months of age in an individual pen by 15 semen collections during the beginning of the breeding season, young bucks are collected twice a week while mature bucks may be collected from 3 to 5 times a week [15]. In order to have more kid crops, producers usually prefer inseminating does using hormone treatment protocols, although such hormone treatment practices are restricted in Europe [16]. Alternatively, male effect may be an efficient way to induce oestrus in an AI program. Insemination could be performed once or twice over a 24-h period after oestrus is detected, such as via introduction of a buck or by the introduction of teaser buck [8]. In the middle of seasonal anoestrus, a high level of fertility (71% to 78% of kidding) was obtained using frozen semen on goats subjected to treatment with artificial long days and progestogen followed by AI 52 h after introduction of the buck equipped with an apron [17].

The seasonal reproduction of bucks inhibits semen collection during 6 months each year, thus limiting the total number of semen doses produced per male during his life. Artificial photoperiodic cycles, extensively used in poultry industry first introduced for rams, permit the control of sexual activity of seasonal breeds. Alternating 1 to 2 months of long days (16 h of light:8 h of darkness) followed by 1 to 2 months of short days (8 h of light:16 h of darkness) diminishes the seasonal variation in sexual activity of bucks [15,18,19]. Different melanizing activity of bucks and ejaculates were first observed in 1964 [20]. It was reported that bucks under photoperiodic treatments for 3 consecutive years and collected twice a week gave an identical fertility rate and higher number of total sperm than test bucks collected twice a week during the sexual season. In another trial with a more intensive collection regime of 4 times/wk during the year, bucks subjected to a treatment of 2 months of long days followed by 2 months of short days yielded more semen doses than bucks kept under the same collection rhythm and natural light from September to February [15,21,22]. Knowledge of the different effects of photoperiod on neuroendocrine pathways and the reproductive activity in goats has therefore allowed producers to successfully apply light treatments/melatonin to control seasonal reproductive activity in field conditions and also in bucks raised in AI centers [23–25]. Photoperiod-treated bucks have the similar capacity as melatonin-treated bucks to induce reproductive responses of female goats during spring [26]. Currently, this photoperiodic scheme is used in France and other European countries in buck stations as part of the goat breeding program. Frozen semen is no doubt beneficial to breeding programs. Semen from a superior buck can be cryopreserved for genetic improvement after progeny testing. Also, fresh semen that is less costly than frozen semen could be used for reproductive management, which is a common practice in developing countries.

## DEVELOPMENT OF ARTIFICIAL INSEMINATION

The modern history of AI begins in 1899 in Russia when professor Ivanow used sponges to recover semen from the vagina of mares following mating. Within a decade, his laboratory was evaluating semen collected from domestic farm animals including horses, cattle, sheep, rabbits, poultry as well as the dog and fox [4,27]. A major advance was made when the first artificial vagina was developed for bulls in 1914, and similar artificial vaginas were also developed for sheep and goats at the same time [4]. In the early stage of dairy goat production, controlled mating was probably considered unnecessary [28]. The AI of cows was used in the early 1930s in Russia, US, and Denmark Until 1960, semen for AI was diluted with a medium called an extender to preserve the sperm’s life. The number of experiments in bull semen conducted in 1960s increased the efficiency of semen by diluting with high proportion and special extenders and freezing in liquid nitrogen. For liquid preservation, goat semen is usually stocked at 4°C. Many extenders for goat semen were tested for liquid preservation but the most efficient one is a skimmed milk-based media [29] or native phosphocaseinate [30]; however, sperm fertility after AI is retained for only 12 h to 24 h and decreases thereafter as the duration of liquid preservation increases. Techniques of production and storage of goat semen based on cryopreservation have been proposed by Corteel in 1974. After separation of seminal plasma from sperm cells to avoid deterioration of spermatozoa, sperm cells are diluted in a skimmed milk-based glucose (0.5 M) and glycerol 7%. Semen is then stored in 0.2 mL straws containing 1×10^8^ sperm cells and deep frozen progressively in three steps into liquid nitrogen (−196°C). It was reported that the deleterious effect of goat seminal lipase is minimized at 4°C when compared with its effect at 37°C or after freezing/thawing [8]. Different practical methods of estrous detection are used. Fertility of goats inseminated once in the 24 h following the beginning of estrous with deep frozen-thawed spermatozoa ranges from 60% to 65% [31]. Due to the low conception rate of goats artificially inseminated with frozen semen, AI using fresh semen has been extensively performed in the dairy goat industry of China, with a conception rate between 75% and 85%.

In the practice, only high quality semen is used to inseminate animals. Initial assessments of each ejaculate involve measurement of the volume and the sperm concentration to estimate the number of sperm collected and a visual assessment of the proportion of sperm displaying progressive motility in a diluted sample at a magnification of 400×. These assessments provide important information on the suitability of the ejaculate for processing, the reproductive performance of the sire, and the number of doses that can be produced and are required for accurate dilution and packaging of semen [32]. Evaluation of which goat semen is useful for AI has undergone changes similar to those for cattle. Assessment pertains to sperm quality including temperature-dependent motility, microscopic visualization of sperm, type of extender, sperm concentration, and time of sperm evaluation etc. Early motion analysis with still images or film eventually led to the development of computer-aided sperm analysis currently in use [4,33]. These systems were developed to provide objective measurement of sperm velocity and to determine the proportion of the sperm population with total and progressive motility. More recently, some units are capable of assessing sperm morphology [32]. It was reported that administration of gonadotropin-releasing hormone (GnRH) analogue (buserelin acetate) during the non-breeding season led to a rapid increase in testosterone concentration and improved sperm quality of bucks [34]. In dairy goats, seasonal variation influences semen quality of Saanen bucks. Fresh ejaculates in summer and autumn with high quality were more suitable for processing to frozen semen. While fresh semen in spring can also be used for AI successfully, the efficiency of goat reproduction could be improved by manipulating the seasonal variation of semen quality for immediate AI and/or cryopreservation [35].

A significant recent change to AI has been the introduction of sex-sorted bull semen based on the amount of DNA difference in the X and Y chromosome [36–38]. Critical to the development of sexed semen technology was the announcement by Johnson et al [38] at the USDA laboratories in Beltsville, Maryland, of the live birth of rabbits, with 94% female pups, from sexed sperm. The work led to a US patent covering the technical details of flow sorting sperm for sex [39]. There are two limiting factors involving the current technology used to sex semen. First, the processing of sexed semen involves flow cytometers that are both expensive to purchase and maintains, and expensive to operate with low efficiency and high costs. Consequently, multiple flow cytometers are used simultaneously to speed up the processing of each ejaculate. The accuracy of selection of either X- or Y-chromosome-bearing sperm is 90%, and the accuracy is closely related to the speed of sorting. Second, the process of sexing sperm results in varying levels of damage to the cells, which is reflected in a decrease in conception rates and embryo production following AI in both dairy heifers and cows compared with unsexed frozen semen [40,41]. Although sperm sexing technology has been found to be effective in many species, no data were available on the DNA content in goat spermatozoa until 2004. The large difference in DNA content between the X- and Y-chromosome bearing buck spermatozoa allows for a clear identification of these two sperm population [42]. The procedures of sperm sorting in dairy goats were first established using flow-cytometry with the purity of 95%. Sixteen goats inseminated with 1.03×106 spermatozoa of frozen sex-sorted semen by intra-uterine laparoscopy had a conception rate of 25% [43,44]. Subsequently, the frozen sex-sorted semen was used in AI for an easy field application, although the conception rate of Xinong Saanen dairy goats with the dose of 4.4×10^6^ spermatozoa was very low at 8.3%, but the results implicated that the conception rate could be improved by increasing the sex-sorted sperm density [44]. However, the high spermatozoa sorting costs and low conception rate of frozen semen insemination render difficult the application of sex-sorted sperm in dairy goat reproduction.

## SYNCHRONIZATION OF ESTRUS

The ES could change the randomness of estrus behavior through the application of exogenous sex hormones so that estrus occurs within a predetermined time. Early investigators have recognized the requirement for synchronization of the estrous cycles in efficient small ruminant production. The use of progestogen sponges and Pregnant mare serum gonadotropin (PMSG) for synchronization of estrus in goats has yielded satisfactory results [45]. Traditional protocols were designed with the aim of controlling the luteal function by exogenous progesterone/progestogens administration for 10 to 14 days (long-term protocols). New protocols for AI achieve a better control of follicular development and ovulation that enhances fertility, mainly by reducing progesterone exposure from 10–14 days to 5–7 days (short-term protocols). This simple strategy avoids the detrimental effect of low progesterone concentrations during long periods when the intravaginal devices are placed for many days. These short term protocols consist of exposure to exogenous progesterone usually in a controlled internal device releasing (CIDR)-type intravaginal device for 5 to 7 days, associated with a dose of equine chorionic gonadotropin (eCG) and prostaglandin F_2α_ (PGF_2α_) at the time of device removal [46]. PGF_2α_ is widely used in ES, which modifies ovarian follicle development, prompts corpus luteum dissolution to resume ovary activity, and contributes to cystic ovary formation [47]. With the advent of prostaglandin, it is possible to synchronize estrus through luteolysis. PGF_2α_ or its analogues have luteolytic function and two injections administered 11 days apart in cycling females give satisfactory results [48,49]. For a treatment length of 11 days, shorter than the luteal phase of 16 days, oestrus and ovulation may be delayed or even inhibited by the presence of a functional corpus luteus at the end of the progestative treatment and cloprostenol must be injected to induce luteolysis [50]. Tests on decreasing cloprostenol doses from 200 to 0 μg indicated that the best fertility after AI was obtained after one intramuscular injection of 50 μg 2 days before sponge removal. Removing sponges 48 h after the PMSG injection gave higher fertility rates (53%) than simultaneous sponge removal and PMSG injection (48%). The PMSG dose is determined according to season, parity, and daily milk production during the month preceding the hormonal treatment. For lactating goats, the current dose is 400 IU and it is increased to 500 IU for goats inseminated before the middle of June. An additional 100 IU is administrated for goats yielding more than 3.5 kg of milk per day in any season. For nulliparous does, 300 or 250 IU doses are recommended for inseminations before or after middle of June [15,51]. The ES combining with AI protocol has been widely used in intensive large-scale dairy farms in the world, which could increase economic income by reducing the management cost [52,53]. The study showed that repeated ES treatment may alter FSH secretion patterns with reduced FSH pulse frequency, thus influencing the estrus response of twice-treated ES goats [54]. However, the efficiency of intrauterine insemination with frozen thawed semen requires a more precise synchronization of ovulation in treated goats. During the breeding season, there are different estrus induction or synchronization schedules available, among them treatments of goats with long-term vaginal progesterone suppositories and injection of FSH or its analogue PMSG have been known to be effective ES or superovulation methods [55]. It was reported that using fluorogestone acetate (FGA) intravaginal sponges short term 5 d and long-term 11 d PGF_2α_ +eCG treatments as well as 5 d GnRH+PGF_2α_ 5 d treatment provide reasonable levels of induction/synchronization of oestrus and fertility after natural mating in lactating goats during the transition period. The combined 11 d FGA+PGF_2α_ +eCG treatment resulted in an efficient method with respect to synchrony of oestrus and ovulation, but it was accompanied by a high incidence of abnormal ovarian response [56]. Different studies of ES reported so far show that short-term protocols using intravaginal progesterone devices result in a series of benefits compared with the long protocols used previously, namely better control of follicular response and ovulation, acceptable pregnancy rates, shorter periods for implementation, and eventually, the possibility of reuse of silicone devices, thus reducing the cost of the treatment [57].

## EMBRYO TECHNOLOGIES

As the major advances were being made in methods of semen collection, extension, and preservation in dairy goats between 1970s and 1990s, there was a parallel research effort focused on females that facilitated the commercialization of AI. Due to the fast development of such technologies in dairy cattle, corresponding similar research in dairy goats occurred at almost the same pace. For females, superovulation and ET are seen as methods for rapid multiplication for superior animals [32]. Although recovery and transfer of embryos from females does not match the enormous potential genetic contribution of individual bucks, it does allow production of more kids per female, which is very beneficial to produce more offspring of high producing does. *In vitro* production of embryos by combining ovum pick-up, *in vitro* fertilization, and ET, embryo culture medium enabled embryo manipulation techniques to develop. It is now possible to freeze embryos, store them for future use or transport them internationally, bypass certain disease situations, initiate production of kids from reproductively immature doelings, and evaluate embryos before transfer.

The sequence of events leading to the ET usually starts with superovulation. The first superovulation in cattle was performed by Casida et al (as cited in Moore et al [32]) in 1943. Dairy goats of all breeds could ovulate 1 to 3 eggs per estrus cycle, but 10 to 20 available oocytes can be obtained from these goats after superovulation treatment with appropriate doses of FSHs [58]. The principle of superovulation is to artificially add exogenous gonadotropin so that more follicles have the opportunity to continue development. Finally these follicles will be able to complete the conversion from FSH-dependent to LH-dependent, and will establish the status of dominant follicle [59]. PMSG and FSH are widely used for superovulation in the dairy goat industry. Primary methods for inducing follicle stimulation and oocyte production are using PMSG or eCG, because of its long half-life, only a single injection of PMSG was required for inducing superovulation in goats and cows. Due to 72 h half-life of PMSG, goat ovaries are prone to appear a large number of anovulatory follicles and corpus luteum of early degenerate after PMSG injection, thus FSH was applied for superovulation of dairy goat [60].

Superovulation with FSH was reported in 1958, and when ET programs changed to porcine FSH, injected twice daily over a period of 4 d, more consistent superovulatory results were achieved [61,62]. However, because the biological effect of FSH is affected by FSH/LH ratio, purification methods and other factors, the FSH should be carefully selected for superovulation treatment. The FSH or other follicle stimulating gonadotrophin administration was initiated on any day starting between d 11 to d 13 of estrous cycle if the day of previous cycle is available. A major problem often associated with superovulation in goats is premature regression of the corpus luteum, reported in up to 27% of donor animals [63]. This phenomenon may be associated with early return to estrus before the normal time for embryo collection. Embryo recovery rates are often reduced and this is thought to be associated with abnormal tubal transport of embryos in does [49]. The embryo production and transfer technology in sheep and goats had made a great progress in 1970s because of the fast development of ET in cattle [64]. Efficacy of PMSG and FSH has been compared for superovulation in goats. The mean ovulation rate was significantly higher in FSH than PMSG treated goats, and the incidence of large follicles that failed to ovulate was higher in PMSG treated than in FSH treated goats. Similar results were obtained for number of transferable embryos [48,65]. It is believed that higher incidence of large follicles failing to ovulate in PMSG treated goat may be due to higher levels of plasma estradiol, which persisted longer in PMSG treated than in FSH treated females, and the endogenous PGF_2α_ of either follicular or uterine origin may possibly be a causative factor in premature luteal regression [49]. Efficacy of norgestomet ear implants, progesterone injections, PGF_2α_, and horse anterior pituitary extract in producing ovarian responses, embryo recovery, and fertilization rate in goats were compared, without significant differences being noted [66,67]. When the superovulation was used in field condition, most producers don’t want the ovulated follicles above the normal range, study showed that an increased dose of PMSG injection significantly increased the numbers, total diameters, and diameter of each dominant follicle and corpus luteum, and injection of 7.5 IU PMSG/kg body weight at the luteolytic stage could be applied to increase the secretion of pregnancy hormones without increasing the number of ovulating follicles and corpora lutea above the normal ranges [68].

The embryo collection was first performed surgically in cattle, which was invasive, expensive, time consuming, and hard work. It required sophisticated surgical facilities and could not be performed on farm. The non-surgical techniques are easier on both ET practitioners and animals, simple, and relatively low in cost. Initial techniques were developed by Rowson and Dowling [69]. Procedures of embryo collection of goats either surgically or non-surgically are similar to those for cattle and have changed little as described by Hunter et al [70]. Successful recovery of embryos has been reported with surgical methods from oviducts or the uterus. Embryos are recovered from donors 4 to 5 days after mating, all donor goats are taken off feed for 24 h and water for 12 h prior to surgery, and the collections are carried out under general anesthesia [48,49]. A mid-ventral line incision is made cranial to the udder attachment to allow elevation of each side of the reproductive tract. The reproductive tract of the animals is flushed with Dulbeccos phosphate buffered saline containing 5% bovine serum and 1% antibiotic-antimycotic [71]. For oviductal flushing, the oviducts are cannulated through the fimbriated end with a fluted plastic catheter. For uterine flushing, a No. 8 pediatric Foley catheter is inserted in the uterine horn just proximal to the bifurcation and its bulb is inflated with air. The flushings are collected in sterile plastic Petri dishes for immediate examination and counting under a dissecting microscope [49]. Care should be taken throughout surgery to minimize postoperative development of abdominal adhesions. There appears to be little effect of collection time after estrus on rate of embryo recovery.

Exteriorization of the reproductive tract at laparotomy for the purpose of embryo collection inevitably involves some degree of surgical trauma and often leads to the formation of postoperative adhesions which may involve the uterus and ovaries. Surgical collection of ova generally makes repeated embryo recoveries from valuable donors an undesirable or difficult task [72]. Non-surgical collection of embryos involves the use of a laparoscope or the transcervical passage of a catheter by mechanical dilatation of the cervix [49,73,74]. Nonsurgical embryo procedures can be performed in a relatively simplified way. Nonsurgical embryo recovery does not require the prolonged fasting period, drug retention is minimized, and donors can stay in a standing position. After the end of embryo recovery, donors are promptly returned to their routine housing and feeding conditions. Promising results using nonsurgical embryo recovery in goats has been reported [75]. The embryos collected usually range in development on those days from late morulae to expanded blastocysts. Microscopic evaluation includes determination of the stage of development, particularly in reference to the day of collection, organization of the embryo, morphological appearance of the blastomeres, degree of fragmentation, and other signs of degeneration [32,61,76]. Embryo recovery rates have improved over the years, and with the development of laparoscopy, it has now become possible to collect embryos from the same female donor repeatedly, with the minimum complication of adhesions. Repeated hormonal superstimulation on the same individual has, however, been reported to lead to a decreased ovarian response due to an immune response by the donor [77].

The successful cryopreservation of sperm first reported in 1949, embryo cells contain far more water than do sperm cells, and effective dehydration of these cells to prevent ice damage is critical to survival [32]. Success with goat embryo cryopreservation was first reported in 1970s by the slow freezing methods for *in vivo* derived embryos, and as with sperm cryopreservation, an optimal cooling rate applies also to embryo cryopreservation [57]. The optimal cooling rate depends on the amount of water in the cells and the ease with which it can leave the cells. Slow freezing is the default method for *in vivo* derived embryos used by many practitioners worldwide, resulting in acceptable embryo cryotolerance and pregnancy rate; however, when slow freezing is applied to *in vitro* produced embryos the outcome is poor. Substantial efforts have been made and some interesting strategies have been proposed to improve the survival rate of *in vitro* produced embryos subjected to slow freezing [78]. Systematic comparisons were made on the effect of cooling rate, warming rate, and the use of different cryoprotective agents. The slow cooling rate gave water sufficient time to exit cells osmotically so that damaging intracellular ice crystals would not form, and removing cryoprotectant from cells postthawing minimized osmotic swelling [79]. Multiple factors are associated with the lower cryotolerance of embryos produced *in vitro* compared with embryos produced *in vivo*, such as excessive cytoplasmic lipid content, changes in the structural, physical and chemical characteristics of the embryo, the stage of embryo development, media composition, and protocols [57]. Since the 1990s, several methods of vitrification have been proposed in goats as an alternative to slow freezing, both for *in vivo* derived and *in vitro* produced embryos. The novel concept of minimum volume vitrification, with ultra-high cooling rates and high media viscosity, has appeared as a renewed hope for progress in embryo cryopreservation in various species, and was evaluated in goats [80–82]. Embryo cryopreservation had showed a large commercial value in the dairy goat industry. The embryos can be collected at one time and place and used as needed with the exact number of recipients required at another time and place with advanced cryopreservation technique. However, even though important advance have been achieved on cryopreservation of *in vitro* produced embryos and oocytes, further investigations and some refinements are still necessary in order to obtain an easy, fast, low-cost and effective method for a wider application of this technology [32,57].

## EMBRYO TRANSFER

The first ET in goat was reported by Warwick et al [83] in 1934. Nineteen 2–16 cell embryos were transferred to 18 recipient ewes, and eight lambs were born [70]. Transfer of *in vivo* or *in vitro* produced embryos to the uterus of the recipient ewes and does can be reliably achieved by means of laparoscopy. The procedure reduces the time taken for each transfer and the conception rate is similar (70% to 75%) to that obtained using mid-ventral laparotomy. In the context of employing non-surgical methods for animal welfare reasons, transcervical ET has been attempted and did not observe any significant difference in terms of kidding between non-surgical and laparoscopic transfer of embryos [84]. However, the pregnancy rate is still too low to justify the routine use of this method [64]. In surgical transfer, recipients are anesthetized as for donor does while collecting the embryos. The reproductive tract is exteriorized through a ventral midline incision to allow visual confirmation of a corpus luteum. Transfers are made to does which have been detected in estrus within 24 h of their respective donors. Each recipient receives one or two embryos ipsilateral to the ovary containing one or more corpora lutea, or to either horn where both ovaries contained corpora lutea [85]. The simplified laparoscopic method can achieve high pregnancy rates and appears to be a safe, minimally invasive surgical procedure. It should be encouraged for the transfer of embryos in goats, and limited studies have reported success for transcervical ET in small ruminants [49]. The first success after transfer of a frozen-thawed embryo was obtained in 1970s using the slow freezing method [86]; the majority of goat embryos are cryopreserved by slow freezing with embryo survival rate ranging from 27% to 59%, considering only excellent and good quality embryos. Embryos were first stored in media containing dimethysulphoxide as a cryoprotectant, but ethylene-glycol has emerged as a superior cryoprotectant with higher survival rates approaching those achieved with fresh embryos and with the possibility to transfer the embryo directly after thawing [87]. Goat embryos are able to survive vitrification procedures, and with further research these methods may provide an economical alternative to the current freezing methods requiring gradual dehydration of embryonic cells. Addition of 0.4 M sucrose in association with direct ET significantly improved the viability of goat vitrified embryos with a survival rate of 34% [88]. Encouraging results have been obtained in terms of survival rates of vitrified-thawed goat embryos produced *in vitro* [64]. The use of ethylene glycol and slow cooling rate was efficient for the cryopreservation of goat embryos, resulting in good embryo survival rate when directly transferred to recipient goats, even after the transfer of lower quality embryos, kidding rate of recipient goats with frozen thawing embryos was 60% [89]. ET is now extensively used in the dairy goat breeding and production program.

Considerable research into *in vitro* embryo production in goats has been undertaken in an attempt to determine what conditions are needed for the three subsequent steps of *in vitro* maturation, *in vitro* fertilization, and *in vitro* culture [90]. These *in vitro* processes attempt to mimic the *in vivo* processes of embryo development in the female reproductive tract. Reviews of the different protocols used for *in vitro* embryo production in goats have been reported in the past [62,91–93].

## GENETIC REGULATION OF REPRODUCTIVE TRAITS

Efficient dairy goat production depends on the quality of breeds and the number of high producing animals. It also largely depends on the goat fertility, which is the ability to reproduce offspring with normal reproductive function. This ability is affected by environment, nutrition, breeding method, and reproductive skills [94]. The rich resources of goat breeds in China include most Southern native goat breeds such as Sichuan Dazu black goats, Jining black goats, Guizhou white goats, Shaannan white goats, etc. and have high fecundity; those breeds play an important role in dairy goat breeding and selection [94,95]. They provide resources for research on the molecular mechanisms and genetic improvement of reproductive traits. Cyclin B2 is dispensable in mammals and activated cyclin-dependent kinase 1 in oocytes. Adenylate cyclase 1 plays a role in oocyte meiotic arrest and resumption. DNA methyltransferases 3b is essential for early development of oocyte. Smad2 is active by transforming growth factor-β related signaling and patterned the embryonic axis, these genes are candidate genes in high fecundity goats population [96]. The genetic polymorphisms of bone morphogenetic protein-15 (BMP15) are reported to be associated with litter size and increased ovulation rate [97,98]. BMP4 helps in endothelial progenitor differentiation and myogenic induction during embryonic development [99,100]. The mechanisms of goat fertility inheritance remained unsolved although many advance sequencing technologies were used to screen the possible genes associated with the reproductive performance of dairy goats.

Goat fertility is an important functional trait, because it is one of the main traits for the selection of small ruminants [101]. China’s goat industry has given much attention to improving goat fertility, such as application of crossbreeding, feeding management, estrus control, AI, and ET, resulted in a considerable economic profits [94]. However, due to the lack of exploration and research in the theory of reproductive physiology and fertility regulation, thus the undesirable breeding outcomes attribute to incomplete and short sighted breeding plan.

## PROBLEMS WITH THE REPRODUCTION OF DAIRY GOATS

The ES techniques have been widely used to induce cyclicity in anestrus dairy goats and synchronize estrus, which produced a uniform kidding times and lactation periods [102, 103]. Appropriate ES protocols can markedly alter the onset, interval, and duration of estrus as well as the rates of pregnancy and kidding [104].

In the dairy goat industry, PMSG+CIDR or FSH+CIDR is most commonly used as effective protocols for ES or superovulation [105]. However, in farming practices, lower reproductive performance is observed in female animals treated with repeated gonadotropin [106]. Studies have shown that too many repetitions of superovulation by PMSG or FSH cause adverse reactions in ovary development as well as reproductive performance. When superovulation is induced more than once for the same animal, the response to the treatment and gamete quality are reduced [107,108]. Decreased fertility rates after repeated gonadotropin treatments are related to an increase in anti-FSH [109] or anti-PMSG antibodies [107]. It is speculated that repeated treatments provoke an immune response that effectively destroys biological activity of gonadotropins, and high concentrations of either anti-PMSG or anti-FSH antibodies in animals lead to ovary refractoriness [110,111].

## PERSPECTIVES

In recent years, the high nutritional quality and health benefits from consumption of goat milk have become more widely acknowledged, people have gradually realized that goat milk has higher nutritional value than cow milk [112]. The dairy goat industry has not yet become well developed in many developing countries, less than half of total goats are used commercially, and there is great potential to produce more milk and develop many high quality milk products. With the deepening understanding of the value of goat milk, the goat milk market will gradually growing and expand, and the dairy goat industry will receive increasing attention and investment. The dairy goat production has several advantages, such as small investment, short reproductive cycle, easy feeding, and quick economic returns. These advantages demonstrated the promising prospects of dairy goat industry. However, the dairy goat industry also faces several challenges, such as lesser number of large-scale intensive dairy farms, limited consumer market of milk products, and fewer high producing breeds of dairy goats etc. Therefore, it is necessary to formulate relevant breeding regulations to form a supporting breeding technique, and it is also very important to accelerate the development of key technologies of reproduction, breeding, and feeding management in the dairy goat industry. These measures could improve the production performance of dairy goats to provide more excellent goat products for human consumption.
